# Women as Workers

**Published:** 1892-09-17

**Authors:** 


### Women as Workers.

### II.—WHERE WOMEN NEED NOT FAIL.

From time to time various charges have been brought against women as workers. They are inaccurate; they are hopelessly unbusiness-like; they have no notion of "honour," as far as public matters are concerned; they can't look at things "in the large"; they never work steadily at anything; they can never leave a thing alone, but keep on bothering themselves and other people about it. These are some of the charges brought against them by their critic and fellow-worker, man. And doubtless there is some amount of truth in what he says. On the other hand, there is a good deal to be said on behalf of woman, in the way of excuse for her shortcomings, and of argument that she could not develop into a full-blown worker all at once, any more than Rome could be built in a day. But as this paper is not an apology for women, but rather a discussion of what they can do, and how they may best do it, it will be more to the point to ask how their failures, where they fail, may be remedied.

True, we may look to time to do a good deal in the way of correcting the faults and developing the powers of women workers. But there is no reason why these workers themselves should wait upon his deliberate steps. If they once see where they are likely to fail—what, in fact, are the "defects of their qualities," they have but to keep watch over themselves, and many of these defects will disappear like dew before the sun. To take at random one of the charges mentioned above—is it true that women lack perseverance? Yes—and no. We must all of us know women who give themselves to their work heart and soul; but doubtless there are also women who show plainly enough that they are only working under compulsion. Still, it does not follow that they will never take to work cheerfully and heartily. Mr. Walter Besant generalises too hastily when he speaks of its being unutterably distasteful to a woman to earn her daily bread. Many women are much the happier for having to do it; and for one who shows that it "goes against her" to work, there are a dozen who find in work their highest happiness. Only—and this is how the charge of want of perseverance has come to be made—they find the time of "breaking in" a very hard one. Comparatively few educated women are brought up to "drudge." They have been used to flit from one task to another, to throw aside the book for the sewing, or both sewing and book for a run in the sunshine; and when they first realise what it means to be cooped up for many hours in the day at one particular task, it seems at first almost more than they can stand. "Well born women," it has been well said, "can work splendidly for a time under any impulse, but if they are obliged to work for eight hours a day continuously throughout the year, they either acquire a new habit from the beginning, or they sicken—physically sicken—of the drudgery, and die, or they abandon such labour in unconquerable disgust."

This, then, is what is wanted—a "new habit." A woman who is about to take up work for the first time must realise that it is the first step that costs—that there will be a time of suffering, and of desperate longing to escape; and she must make up her mind that, for her own good, and for the credit of herself and her sex, she will live this time through. The breaking-in is painful, but it does not last for ever; and it would be an excellent thing if those who look forward to a life of hard work, whether undertaken for love or for money, would prepare themselves for this breaking-in by putting a check upon their own sweet wills, and teaching themselves, while yet they are free agents, something of this new habit.

And what of the charges that women cannot look at things "in the large," and that they have no notion of honour? Well, here, again, time will do much, and so can the delinquent herself. Women have always been trained to attend to details, and prove themselves, moreover, remarkably apt at the work; and it is no wonder if now and then they lay themselves open to the charge of "not seeing the wood for the trees." But the more they are occupied with larger things, the less petty will their minds become; and meanwhile they should recognise the danger, and do their best to avert it, by drawing off now and again, and taking a look at their work as a whole. Much the same may be said as to their defective sense of honour in dealing with public or business affairs. "Where the knowledge and practical experience of women are defective," says Miss Edith Simcox, "so also is their sense of moral obligation. The intellect of women, as a class, has been concentrated and expended upon the incidents of private life and the domestic relations, and within these limits, as a natural consequence, their sense of moral obligation has been developed." Doubtless, there will always be mean business women—as there are mean business men; but on the whole the introduction of women into business affairs should mean an increase, rather than a decrease, in morality, when once they have felt their feet, and brought to bear all their powers, both of mind and soul, upon it.

Again, as women get more used to work, they will throw off the tendency to be "careful overmuch." It is more difficult for a woman than for a man to keep business worries for business hours, owing to the greater sensitiveness of her nerves, but she must learn to do it if she wants to live long and to work well. No one can make a good nurse who lies awake at night thinking over her "cases," or carries the memory of a terrible operation with her to the dinner-table; nor can any man or woman be a permanently good worker who brings the week into the Sunday, or the office into the recreation-ground. Mr. Gladstone probably owes as much to his power of throwing off worries, as to his magnificent physique; and those who charge him or any other statesman with levity because he is seen, during some time of anxiety, at a theatre or cricket match, are doing a thoughtless and a cruel thing. Women, then, must learn how to forget. There must be some time for serious thought, or the details will usurp the place of the whole, but then both whole and details must be locked away out of sight until the next business hour comes round.

Women workers, as we at present know them, fall into two classes—those who spare themselves too little over their work, and those who spare themselves too much—which is but of a piece with the truth that when a woman is good she reaches a higher level than a man, and when she is bad she falls to a lower. Very few men throw themselves into their work with such utter devotion as do the highest class of women-workers; while as for the others—well, woman is not so unlike the little girl of nursery fame, who, "When she was nice, was very, very nice; and when she was not, was horrid!"

As for inaccuracy and unbusiness-likeness, the other faults with which women are charged, they must be sternly battled against. "Whatever is worth doing is worth doing well," is a maxim that sadly needs to be impressed upon some women. Miss Florence Wilford has lately addressed some very sensible words to "Limp Ladies," that is, to ladies who know how to do nothing well, and whose feeble attempts at work are the despair of their friends and the laughing-stock of their acquaintances; and, after all, there is no reason for this limpness, except that some women seem to "love to have it so." It is not so much a good education as self-culture, the keeping of one's eyes and ears open, and the determination to learn what is to be learned even from the little things of life, that really makes a capable man or woman. To some this habit of mind comes naturally, but all can cultivate it; and there are few lines of life indeed in which general information, and a mind sharpened and widened by the habit of picking it up, are not of value.

